# Wilson’s Disease and Ulcerative Colitis in the Same Patient: Just A Coincidence? A Case Report and Literature Review

**DOI:** 10.4021/gr271w

**Published:** 2010-11-20

**Authors:** Filipe G. Nery, Irene Marques, Marina Magalhaes, Helena P. Miranda

**Affiliations:** a Medicine Service, CHP – Hospital Sto Antonio, Porto, Portugal; b Neurology Service, CHP – Hospital Sto Antonio, Porto, Portugal

**Keywords:** Wilson’s disease, Ulcerative colitis, Association, Copper

## Abstract

Ulcerative Colitis (UC) is a chronic relapsing inflammatory bowel disease (IBD). Wilson’s disease (WD) is a disorder of copper (Cu) metabolism due to inherited mutations in a gene encoding a putative Cu-transporting P-type ATPase, with a heterogeneous clinical presentation that includes hepatic, neurological, or psychiatric symptoms. The case of a 17-year-old female that presented with severe liver failure, three years after UC onset, and in which diagnosis of WD was established is reported. We review the literature and discuss the possible association between the two rare diseases. Although evidence of a common genetic background between UC and WD has not been described, high Cu serum level is present in both diseases. Cu is one of the trace elements necessary for antioxidant defenses during inflammatory processes, affecting the production of free radicals of oxygen and the levels of cellular antioxidants. The presence of both entities in the same patient may suggest abnormal metabolism of Cu or be just a coincidence.

## Introduction

Wilson’s disease (WD) is a rare genetically autosomal recessive inherited disorder of copper (Cu) metabolism that affects mainly children, adolescents and young adults. The ATP7B gene mutations (in chromosome 13) [1, 2] result in an inadequate excretion of absorbed dietary Cu via bile and in the accumulation of toxic amounts of Cu in liver and other organs [3, 4]. The diagnosis of WD is based on clinical and laboratory findings, including low serum ceruloplasmin (CP), increased urinary Cu excretion and hepatic Cu content [5]. In recent years, the development of new techniques in genetic and molecular biology has provided useful tools in the diagnosis of WD [6]. Ulcerative colitis (UC) is a rare inflammatory bowel disease that affects colorectal mucosa. Genetic susceptibility has been described, namely HLA antigens class II [8], and genomic linkage to chromosomes 3, 5, 7 and 12 [7]. The diagnosis takes into account clinical, endoscopic and radiologic findings, as well as histological features.

The clinical association between UC and autoimmune liver diseases, such as primary sclerosing cholangitis [8-10] and hepatitis [11] is well known, though the association with WD is very uncommon.

## Case Report

A 17-year-old female was diagnosed with UC, based on characteristic clinical and endoscopic findings: bloody diarrhea, hyperemia and friability of the colonic mucosa with scattered erosions up to 20 cm from the anal margin, supported by histological features.

She was treated with mesalazine and corticosteroid during relapses. Three years after UC onset, she was admitted to another hospital with a two-month history of nausea, vomiting, jaundice and abdominal pain. Fever, clinical signs of encephalopathy or ascitis were not present. Laboratory data at admission showed increased aspartate transferase (AST = 113 IU/L; N < 32), gamma-glutamyl transferase (GGT = 223 IU/L, N < 39) and total bilirubin (TB = 21.94 mg/dL, conjugated bilirubin of 11.19 mg/dL). Alanine aminotransferase was slightly increased (ALT = 32 IU/L; N < 28) and alkaline phosphatase (AP = 21 IU/L; N < 104) was within normal range. Serum albumin was decreased (Alb = 2.4 g/dL; 3.5 < N < 5.0), prothrombin time prolonged at 6.7 seconds and the platelet count was normal. Hemoglobin was 9.6 g/dL, and she was Coombs negative. She presented a leukocytosis (WBC = 13.1 x 10^3^/µL; 4.1 < N < 10.9 x 10^3^/µL) with neutrophilia (8.69 x 10^3^/µL). Serologies for hepatitis A, B and C were negative as well as for HIV1 and 2, EBV, CMV and toxoplasma. Anti-nuclear antibodies, anti-LKM, LC1, SLA/LP, smooth muscle and anti-mitochondria were all negative. Only atypical PANCA (X-ANXA) was positive in a titer of 1/80. Alpha1-antitrypsin (241 mg/dL; 90 < N < 169 mg/dL) and C reactive protein (1,28 mg/dL; N < 0,50 mg/dL) were elevated. Due to the clinical suspicion of cholangitis, antibiotherapy with piperacillin and tazobactam was started. Two days later, her health condition deteriorated and she was referred to our hospital. Total bilirubin increased up to 57.4mg/dL and prothrombin time was prolonged over 16 sec. An upper abdominal ultrasound examination showed a discrete hepatomegaly (16 cm), with normal echostructure and a normal biliary tree. A transjugular liver biopsy revealed portal and perisinusoidal bridging fibrosis, with moderate inflammatory activity, compatible with cirrhosis. The colonoscopy was not repeated.

The diagnosis of WD was established, based on the presence of Kayser-Fleischer rings in the slit-lamp examination, a low serum ceruloplasmin (CP = 0.11 g/L; 0.18 < N < 0.53), elevated 24-h urine Cu (24h U Cu = 128.128 µmol/d; 0.040 < N < 0.550), and elevated hepatic tissue Cu (TCu = 1112.51 µg/g dry weight; 10.00 < N < 35.00). Neurological examination and cerebral MRI were normal. Genetic testing confirmed the diagnosis Allele1: c.3207C>A(p.His1069Gin) and Allele 2: c.3061-12T(IVS14-12T>A. She was initially treated with trientine (250 mg/day) and zinc acetate (50 mg bid), resulting in progressive clinical and liver function improvement, and was discharged home two weeks after the initiation of treatment. Nowadays, with a follow-up period of three years, she is still under trientine and mesalazine therapy and asymptomatic, without any documented UC exacerbation while on Cu chelation therapy. During the follow-up period she had a pregnancy that progressed uneventfully.

## Discussion

We report a new case of UC associated with WD, the fifth that we are aware since the first case ever reported in 2002 by Torisu Takahiro [12]. All the cases previously described, reported UC complicating previous WD established diagnosis [13, 14]. Our case report describes the association, only, for the first time, in the opposite direction: a case of WD preceded of UC diagnosis.

The possible association between the two diseases was discussed by Torisu et al, considering that Cu acts as a scavenger of oxygen-free radicals during the inflammatory process. The Cu overload in the tissues induces excessive oxidative stress which affects the liver (contributing to inflammation followed by fibrosis and cirrhosis) and the gut (namely the colonic mucosa), where the reactive oxygen intermediates (ROI) helps the promotion of chronic intestinal inflammation and immune activation [12].

Although human Cu metabolism is not completely understood, it is known that blood and tissue concentrations of Cu increase dramatically in WD, in inflammation and in other known conditions [15]. In patients with UC (either active or in remission) there is evidence of high serum levels of Cu and zinc, related to acute phase proteins as well as to hematological parameters of relapse [16]. As Torisu et al described, and as confirmed by other studies, proteins containing Cu and zinc used by the organism as scavengers of ROI, such as superoxide dismutase and metallothionein, are present in decreased levels in the colonic mucosa of patients with active UC and may have a role in the pathogenesis of the disease [12, 17].

So, a correlation may be established between high Cu levels in UC and in WD. UC relapses could also be a consequence of high levels of Cu induced by WD.

In our patient, abdominal pain, leukocytosis and elevated acute phase proteins may suggest a concomitant UC exacerbation during acute liver disease. It is known that high levels of alpha1-antitrypsin could be correlated with UC exacerbation, and C reactive protein and blood leukocytes are noninvasive markers for endoscopically active disease [18]. Nevertheless, at that time, no colonoscopy or colorectal mucosa biopsies were made to confirm such hypothesis.

Copper chelation in patients with UC and high Cu levels may be considered as a part of therapy, but further studies are needed to confirm their role in the course of UC. This implies that UC would appear as a consequence of a silent WD.

Even though WD and UC are both genetic diseases, different chromosomes are implied. In spite of that there are genetic mechanisms that control copper metabolism and homeostasis, related to copper absorption in many of the mammalian tissues, namely in the enterocyte, controlled by Ctr1 (a plasma membrane copper transport protein) [19]. It seems that copper transporter Ctr1 is downregulated when Cu is overloaded in the hepatocytes in WD [20]. If so, the downregulation of Ctr1 expression could lead to an augmentation of intestinal Cu content and inflammatory exacerbation, but a relationship between this transporter and inflammatory bowel disease has not yet been described.

Given the rarity of the reported association between these two diseases, we cannot refute that it could be merely a coincidence, in spite of the mechanisms explained before.

In conclusion, this is the fifth case report published worldwide of a patient presenting with both UC and WD, two rare entities. It seems that Cu plays a central role in both diseases, contributing to fibrosis and cirrhosis of the liver and probably to the immune activation in UC.

We hope that this report elicits further studies concerning the mechanisms associating UC and WD, and stimulates the report of new cases, to allow its real prevalence to be established.
